# Constructing Biological Pathways by a Two-Step Counting Approach

**DOI:** 10.1371/journal.pone.0020074

**Published:** 2011-06-01

**Authors:** Hsiuying Wang, Henry Horng-Shing Lu, Tung-Hung Chueh

**Affiliations:** Institute of Statistics, National Chiao Tung University, Hsinchu, Taiwan; Instituto de Biología Molecular y Celular de Plantas, Spain

## Abstract

Networks are widely used in biology to represent the relationships between genes and gene functions. In Boolean biological models, it is mainly assumed that there are two states to represent a gene: on-state and off-state. It is typically assumed that the relationship between two genes can be characterized by two kinds of pairwise relationships: similarity and prerequisite. Many approaches have been proposed in the literature to reconstruct biological relationships. In this article, we propose a two-step method to reconstruct the biological pathway when the binary array data have measurement error. For a pair of genes in a sample, the first step of this approach is to assign counting numbers for every relationship and select the relationship with counting number greater than a threshold. The second step is to calculate the asymptotic p-values for hypotheses of possible relationships and select relationships with a large p-value. This new method has the advantages of easy calculation for the counting numbers and simple closed forms for the p-value. The simulation study and real data example show that the two-step counting method can accurately reconstruct the biological pathway and outperform the existing methods. Compared with the other existing methods, this two-step method can provide a more accurate and efficient alternative approach for reconstructing the biological network.

## Introduction

One great challenge of postgenomic research is to explore complex biological pathways from genomic data such as DNA sequences, protein sequences, and gene expression profiles. The network building method is widely used throughout biology to reconstruct complex biological pathways.

**Figure 1:**
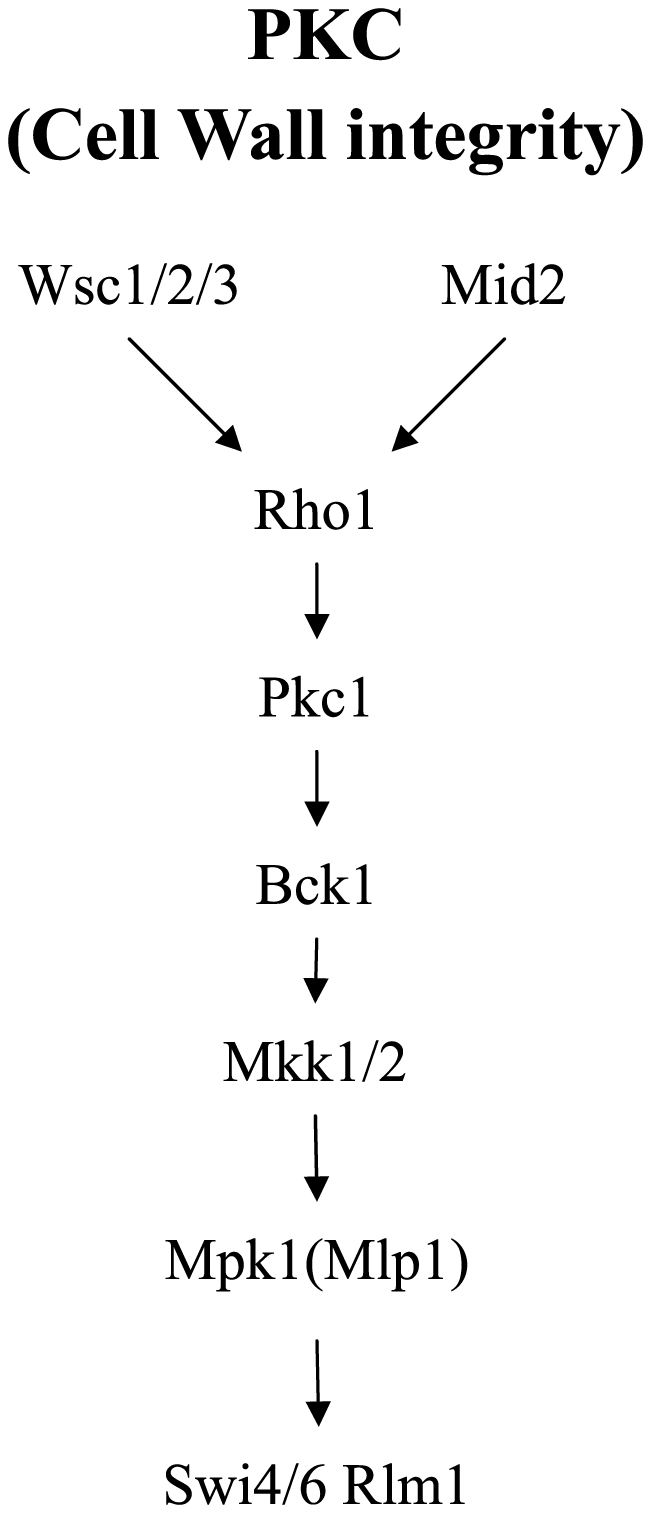
The PKC pathway in yeast. This figure is redrawn from Figure 1A in [1].

We take MAPK pathway as an example. The MAPK/ERK pathway is a signal transduction pathway that couples intracellular responses to the binding of growth factors to cell surface receptors. Robert *et al.* [1] and related studies [2]–[5] based on biology experiments provide the MAPK pathway (Figure 1).

It would be interesting if Figure 1 can be reconstructed in terms of their expression profile of Wsc1/2/3, Mid2,…, etc. To reduce the cost of experiments, one possibility is to predict the activation status of these genes through their microarray expression data for inferring the pathway.

There have been methods proposed in literature for reconstructing genetic regulatory networks in terms of microarray data. For instance, the Bayesian network model is an important technique that has been studied in the last two decades [6]–[8]. In addition, Wei and Li [9] proposed a hidden spatial-temporal Markov random field model to identify genes that are related to biological pathway. Allocco *et al.* [10] provided a variety of methods to find the gene-pairs with similarity relationship. Moreover, other algorithms using linear models [11], [12], differential equation [11], [13], neural network [14] and structural equation modeling [15] have been proposed to explore gene regulatory networks based on genomewide data. However, most of these methods have limitations in dealing with large-scale gene regulatory network because of their complex model structures. Also, careful discretization can be used to denoise high-throughput data. One such example can be found in Xing and Karp [16].

To overcome the disadvantage of the mentioned methods, we consider a simple model based on the Boolean network to reconstruct a large scale gene network in this study. Boolean networks have been proposed and investigated for a long time in literature. Kauffman [17], [18] considered a dynamic version of Boolean networks. Liang *et al.* [19] proposed the algorithm REVEAL to infer gene regulatory network by calculating the Shannon entropy. Akutsu and Miyano [20] proposed an identification algorithm to reconstruct the Boolean network by comparing the collected data with all possible Boolean functions and input datasets. In order to make Boolean network more comprehensive, Shmulevich *et al.* [21] proposed the model of probability Boolean network (PBN). Moreover, for large-scale gene regulatory networks, Kim *et al.* [22] have used Boolean network with chi-square test on the yeast cell cycle microarray gene expression datasets. Markowetz *et al.* [23] proposed the nested effects model to infer the genetic network. Li *et al.* [24] made a comparison between the approaches of probabilistic Boolean network and dynamic Bayesian network. More recent developments are referred to Ay, Xu and Kahveci [25] and Davidich and Bornholdt [26].

In this article, we consider the directed acyclic Boolean (DAB) network as a tool for exploring biological pathways. Our goal is to construct a DAB network from the noisy array data. Since it involves noisy data, the reconstruction of the pathways cannot employ a deterministic inference. Instead, we need to establish a statistical model to capture its random characteristics. A DAB network is characterized by two kinds of pairwise relationships: similarity and prerequisite. The former represents a pair of elements with coherent on-off states. The latter is a partial order relationship, namely, the on-status of one element is a prerequisite for the on-status of another element. More specifically, if one element is a prerequisite to another element, the off-status of one element will restrict another element's off-status. A DAB network is uniquely determined by its state space: all possible on-off states subjected to the pairwise relationships.

Recently, a Boolean implication network is proposed with similar aspect as the DAB network, which investigates all Boolean implications between pairs of genes for large-scale genome microarray datasets [27]. For any pair of elements, they use two statistics to test whether there is any specific relationship between the pair of elements. However, the methods are more applicable for dealing with mass information of datasets.

The approach of building a DAB network based on the expectation-maximization (EM) algorithm to derive the maximum likelihood estimator [28] for a statistical model is established in Li and Lu [29]. Their strategy is to build up a statistical model with measurement error and assign scores for the possible relationships between two genes, and then use the scores to select the true relationship. This method involves more computation and cannot provide a simple closed-form statistic to recover the true relationships between genes.

In this study, we propose a simple method to estimate pairwise relationships between elements from noisy array data. The approach is based on two steps: the first one is to count the numbers of different pairwise relationships in a sample, and the second one is to test the relationship hypotheses according to their asymptotic p-values. Compared with the Li and Lu [29] method, this new approach has a simple closed form and it is not time-consuming. In addition, the proposed counting approach shows substantial improvement compared to the Sahoo *et al.* [27] method. We conduct a simulation study to an example used in Li and Lu [29]. It is shown that the proposed method can recover all of the true relationships. A simulation study for a larger scale network is given in the supplementary material. In addition, the proposed method is implemented on the MAPK pathway example. It can recover 6 true relationships among seven relationships, however, Li and Lu's method only recovers one true relationship in this example. In this real data example, the new method shows a significant improvement in adopting a DAB network for exploring the pathway.

## Methods

**Figure 2:**
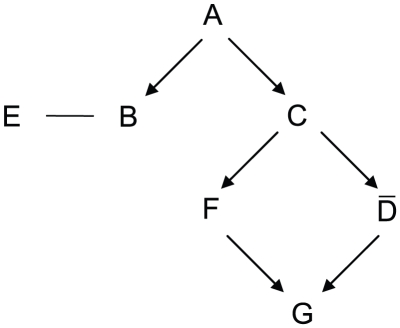
Diagram of a directed acyclic Boolean network with seven elements and twelve pair relationships. Only arrows between covering pairs are shown.

To describe the model and notations, we adopted a simple example used in Li and Lu [29] to illustrate the model assumption. Figure 2 shows the relationships of the seven elements in this example derived from the 13 states of Table 1. In the diagram, the notation 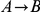 denotes that A is a prerequisite of B and the notation 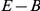 denotes that B and E are similar. Note that 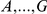 in Figure 2 are called elements. The definitions of prerequisite and similar relationships for any two elements 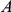 and 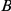 are defined as follows.

**Table 1:** The table of states for directed acyclic Boolean network shown in Figure 2.

case	1	2	3	4	5	6	7	8	9	10	11	12	13
A	0	1	1	1	1	1	1	1	1	1	1	1	1
B	0	0	1	1	1	1	1	1	0	0	0	0	0
C	0	0	0	1	1	1	1	1	1	1	1	1	1
D	1	1	1	1	0	1	0	0	1	0	1	0	0
E	0	0	1	1	1	1	1	1	0	0	0	0	0
F	0	0	0	0	0	1	1	1	0	0	1	1	1
G	0	0	0	0	0	0	0	1	0	0	0	0	1

Assume that an element only has two levels, on or off. We use “0” and “1” to represent “off” and “on” states respectively. For two elements 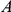 and 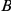, 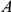 is a prerequisite for 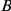 if the on-state of 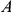 is necessary for the on-state of 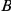, and we denote it by 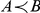. When 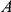 and 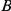 are on and off simultaneously, the relationship between 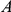 and 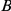 is called similar and is denoted by 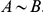 We define 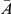 to be the dual state of 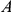. It means that 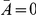 when 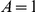.

There are 4 possible situations for the prerequisite relationship, and 2 possible situations for the similar relationship, see Table 2. Totally, there are 6 possible relationships for any two genes. The prerequisite relationship is a partial order. It is transitive on the ground-set, namely, 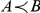 and 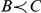 implies 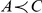. The notations “
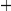
” and “
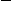
” in Table 2 denote the possible states of 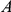 and 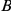 and the impossible states of 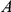 and 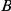 under the relationship, respectively.

**Table 2:** Patterns for the six pairwise relationships assuming exhaustive sampling and no measurement error.

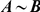			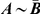			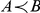 , 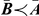		
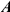 / 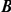	0	1	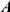 / 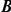	0	1	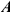 / 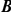	0	1
0	+	−	0	−	+	0	+	−
1	−	+	1	+	−	1	+	+

Let 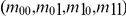 and 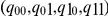 denote the counts and probabilities corresponding to states 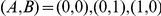 and 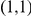 without measurement error. From the possible relationships shown in Table 2, we can propose a hypothesis corresponding to each relationship. For example, the first similar relationship in Table 2 is 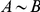, which means that the two situations 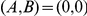 and 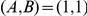 hold. In this case, the probability of the two situations, 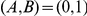 and 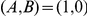, should be zero. Thus, its corresponding hypothesis is 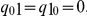 Other situations follow a similar argument. The hypotheses for the 6 relationships are presented in Table 3. Under the measurement error model assumption, let 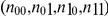 and 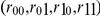 denote the counts and probabilities corresponding to states 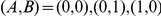 and 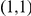 with misclassification probability 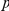.

**Table 3:** The six pairwise between the two elements 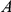 and 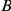.

	Relationship	Hypothesis
diagonal	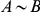	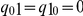
similarity	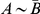	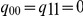
	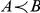	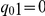
triangular	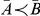	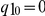
prerequisite	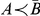	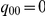
	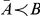	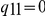

Because of the misclassification error, 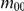 may be split up into four categories. We use the notations 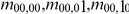 and 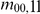 to represent the counts of four cells split from 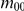. Analogous notations are defined for 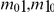 and 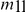. Consequently, their generating probabilities are calculated as follows: 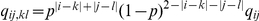. Here, we adopt the notation 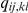 analogous to 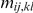. The splitting counts and probabilities implied by misclassification error are given in Tables 4 and 5.

**Table 4:** Splitting counts caused by misclassification error.

A/B	0		1	
0	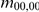	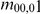	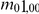	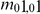
	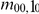	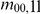	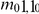	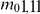
1	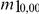	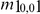	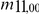	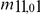
	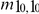	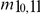	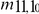	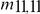

**Table 5:** Splitting probabilities caused by misclassification error.

A/B	0		1	
0	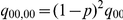	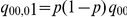	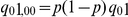	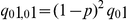
	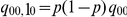	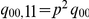	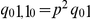	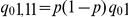
1	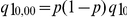	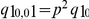	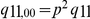	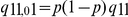
	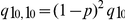	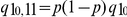	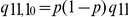	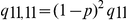

Now we go back to the example of Figure 2 which includes 7 elements. There are a total of 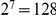 states for a seven-element network. Only thirteen of these states in Table 1 are compatible with the twelve pairwise relationships in the above example. From Figure 2, there are 12 true relationships between the elements, which are
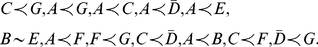
(1)


Under the measurement error model assumption, we do not directly observe the 13 states but observe states with measure error. We aim to reconstruct the true pathway. A proposed method is given in the following.

### The two-step counting method

Suppose we have a sample 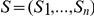 of size 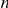 for 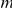 genes where 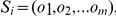 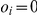 or 1. For example, in Table 1, there is a sample of size 13 for seven genes. We propose a two-step approach to recover their relationships.

### The first step: counting

For a pair of genes, say 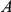 and 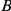, we can count the numbers for 6 relationships in Table 2 for the 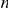 states. The relationships with a counting number greater than a given threshold are regarded as potential relationships.

If there are no measurement errors, it is reasonable to expect that the counting number of two elements, say 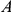 and 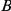, satisfying the true relationship is exactly equal to 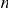. However, since it involves measurement errors, the counting number with respect to the true relationship may not be exactly equal to 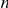. For each pair of elements, we count the numbers satisfying the 6 relationships respectively, say 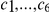. Since we expect that the misclassification probability is low, the counting number(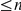) corresponding to the true relationship should be close to 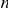. Thus, we can select the relationships with a counting number greater than a threshold. The threshold selection is suggested as follows.

#### Threshold Selection

The suggested thresholds for the similar and prerequisite relationships are
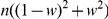
and
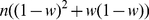
(2)respectively, where 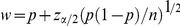. Here, the misclassification error probability 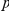 can be assumed to be known from empirical experiences. If 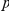 is unknown, the maximum likelihood approach for estimating 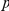 is given in Appendix D in the materials section.

The argument for the threshold selection is given in Appendix A in the materials section. It is based on a confidence bound approach associated with the counting number formulas. The approach is to derive the formulas for the two kinds of relationships, and then uses a confidence interval approach to obtain a lower bound for the counting number formulas. The forms 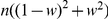 and 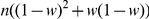 are inferred by the counting number formulas with misclassification probability 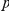, where 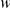 value is derived by a confidence bound approach.

### The second step: asymptotic p-value

Besides directly counting the relationships' numbers, the second step is to test the relationships in Table 3 using an asymptotic p-value. Then we combine both steps to estimate the true relationship between two elements.

The following simulation study shows that the two steps are both essential for selecting the true relationship. If any one of the steps is used solely in selecting the true relationship, the simulation shows that it cannot select the true relationships very accurately.

The p-values derived for the 6 hypotheses with misclassification probability 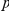 corresponding to the 6 relationships are listed as follows. The derivations are given in Appendix B in the materials section.

For testing 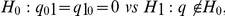 the asymptotic p-value for large sample size 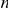 is
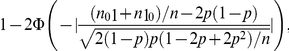
where 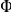 is the cumulative distribution of the standard normal random distribution. The asymptotic p-value for testing 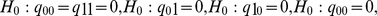 or 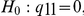 are the forms of (10), (13),(14), (15) and (16), respectively, which are given in Appendix B in the materials section.

The extremeness of the observed value for the test statistic under the null hypothesis leads to a small p-value, which would imply rejection of the null hypothesis. Thus, if the null hypothesis is the true relationship, we expect to obtain a higher p-value. In the second step, we also set a threshold for the asymptotic p-value such that the relationships with asymptotic p-value greater than or equal to the threshold are selected.

A large p-value indicates a larger possibility that the null hypothesis holds. Note that the p-value is less than or equal to 1. In this study, we use the threshold 1 for the p-value criterion in the examples because the largest p-value for each relationship is one. From the simulation study and the real data example discussed in this study, setting 1 to be a threshold for p-value criterion can lead to very accurate results. Note that for other examples, it is possible that the largest p-value is not 1. In this case, we need to observe the p-values to select a suitable threshold.

It is worth noting that the hypothesis testing procedure corresponds to a confidence interval approach [30]. From the confidence interval viewpoint, when the p-value is large enough (close to 1) or small enough (close to 0), we have confidence to accept or reject the null hypothesis. Therefore, in this study, when p-value is 1, we have confidence to accept the null hypothesis.

The two-step method is described as follows.

### Procedure for selecting the true relationship of 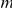 elements

#### Step 1

For a sample of size 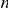 for 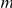 elements, calculate the counting numbers for the 6 relationships of each pair of the elements. Set a threshold for the counting numbers. Select the relationships with a counting number greater than the threshold.

#### Step 2

For each pair of elements, derive the asymptotic p-values for each relationship and set a threshold for the p-value. Select the relationships with a asymptotic p-value greater than or equal to the threshold.

#### Step 3

For each pair of elements, select the relationships satisfying both criteria of **Step 1** and **Step 2**. This relationship is the estimated relationship for the two elements.

Note that it is possible to have more than one relationship satisfying both criteria for two elements. But from a simulation result and a real data application, it shows that in most situation, there is only one relationship satisfying both criteria.

The asymptotic p-value has a closed form which can be easily calculated and the counting number can also be easily calculated. This shows that this method can provide a convenient way to recover the biological pathway.

### An Example

We revisit the example of Figure 2 to illustrate the counting step. Assume that we only have a sample of the states for the 7 elements and we want to recover the 12 true relationships. Note that there are totally 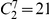 pairs of the 7 elements and there are only 12 pairs with relationships in this example. When considering the case without measurement error, we can reconstruct the pathway from a sample using the counting number method if the sample size is large enough. We can construct the Boolean network for the example by identifying prerequisite or similar relationships. From Table 1, we list the relationship corresponding to the highest counting number for each pair as follows:
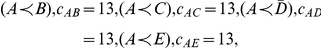


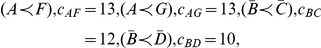


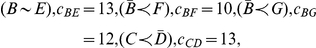


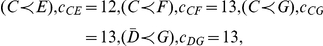


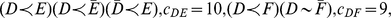


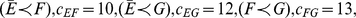
where 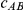 denotes the counting number corresponding to the indicating relationship 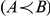.

If there is only one relationship corresponding to the highest counting number, we list that one, such as 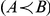; if there are more than one relationship corresponding to the highest counting number, we list all of the relationships, such as 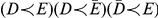. In the 21 pairs, the relationships corresponding to the highest counting number 13 is the 12 true relationships, and the relationships with the counting number less than 13 are not the true relationships.

### Comparison

We consider two existing methods for detecting the pairwise relationships between any two elements. A simulation study is conducted to compare the proposed method with the existing methods for the measurement error case.

### Existing methods

Li and Lu [29] proposed the directed acyclic Boolean network to recover the genetic network. For any pair of element, they use the EM algorithm to calculate the maximum likelihood estimator of misclassification rate 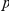 under the multinomial distribution model structure and adopt a criterion that requires a true relationship to correspond to a small estimator of 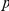 in order to select a relationship. Besides the disadvantage that the EM algorithm is time-consuming, this method is also shown to be less accurate than the counting method from a simulation study.

Another method for inferring the relationship of any two elements is proposed by Sahoo *et al.* [27]. For any two genes 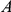 and 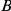, let 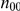, 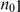, 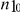 and 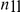 denote the numbers of the four states 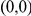, 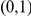, 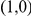 and 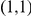 of 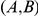, respectively from a sample. For example, to infer whether the relationship 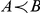 is true, they use the following two statistics to test if the relation 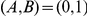 is true:
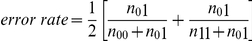


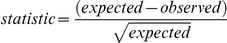
where “
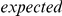
” and “
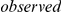
” denote the values of 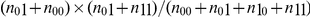 and 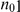, respectively.

The relationship 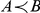 in Sahoo *et al.* method is regarded as true when the “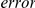 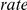
” value is less than 0.1 and “
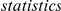
” value is greater than 3 [27]. However, from our calculation, the method may lead to inaccurate results when the sample size is not large. For instance, suppose the number of experiments we observed is 91 and the numbers of states corresponding to (0,1), (0,0), (1,0) and (1,1) are 1, 30, 30 and 30 respectively, resulting in a small “
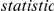
” value of 2.94. Note that the state 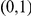 indicates that the relationship 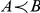 does not hold. However, since the state 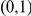 only occurs once, it may be due to a measurement error. In this case, the method does not select the relationship 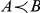. This shows that the criterion is too conservative to select a potential relationship when the sample size is not large enough.

### Simulation

We conduct a simulation study using the example of Figure 2 with 13 compatible states (Table 1) in order to compare the proposed method with the two existing methods. With a misclassification probability 0.05, we generate 100 states for the simulation comparison. Tables 6 and 7 show the counting numbers and p-values for different relationships with a sample size of 100, respectively. Note that the notations 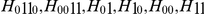 in Tables 6 and 7 denote the relationships in order in Table 3.

**Table 6:** The counting numbers for the 21 pairs in the 100 states under each relationship.

hypothesis	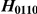	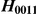	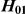	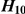	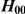	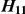
(A, B)	55	45	98	57	90	55
(A, C)	72	28	95	77	93	35
(A, D)	51	49	93	58	95	54
(A, E)	56	44	98	58	90	54
(A, F)	54	46	98	56	90	56
(A, G)	26	74	99	27	89	85
(B, C)	51	49	64	87	83	66
(B, D)	46	54	70	76	77	77
(B, E)	91	9	95	96	52	57
(B, F)	51	49	76	75	71	78
(B, G)	53	47	92	61	55	92
(C, D)	35	65	76	59	94	71
(C, E)	50	50	86	64	84	66
(C, F)	72	28	98	74	72	56
(C, G)	42	58	98	44	72	86
(D, E)	43	57	74	69	79	78
(D, F)	37	63	72	65	81	82
(D, G)	37	63	87	50	66	97
(E, F)	50	50	76	74	72	78
(E, G)	52	48	92	60	56	92
(F, G)	66	34	98	68	48	86

**Table 7:** The p-values for the 21 pairs in the 100 states under each relationship.

hypothesis	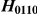	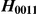	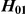	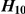	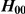	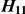
(A, B)	0.8207	0.0065	1	0	0.1358	0
(A, C)	1	0	1	0.0095	0.0680	0
(A, D)	0.1330	0.1511	0.7931	0	1	0
(A, E)	0.9228	0.0046	1	0	0.1201	0
(A, F)	0.7200	0.0092	1	0	0.1527	0
(A, G)	0	0.6534	1	1	0.7599	0.3630
(B, C)	0.9149	0.4654	0.0060	1	0.7636	0.0152
(B, D)	0.4237	1	0.6569	0.6504	0.9855	0.9855
(B, E)	1	0	0.8094	1	0	0
(B, F)	0.9994	0.8352	0.9990	0.9065	0.9118	0.9189
(B, G)	0.4376	0.1168	1	0	0	0.8556
(C, D)	0.0004	1	0.0268	0	1	0.0025
(C, E)	0.7452	0.6280	1	0.0058	0.9122	0.0165
(C, F)	1	0	1	0.0005	0.0014	0
(C, G)	0.0724	0.1689	1	0	0.0070	0.3746
(D, E)	0.1613	1	0.4171	0.4297	1	0.9031
(D, F)	0.0093	1	0.1226	0.1554	0.8975	1
(D, G)	0.0003	1	0.0377	0	0.0001	1
(E, F)	0.9367	0.9716	0.9702	0.7834	0.9965	0.9993
(E, G)	0.3662	0.1523	1	0	0	0.9043
(F, G)	1	0	1	0.0001	0	0.0110

In this case, the maximum value for the counting number is 100 because the sample size is 100. As discussed in above, we can set a threshold (2) for the counting number. In this case, the thresholds for the similar and prerequisite relationships are 86 and 93. And we set the threshold for the p-value to be 1 because the highest p-value for each pair is 1 in this case. The relationships corresponding to the hypotheses with a p-value 1 are the candidates for the true relationship.

For any pair of the 7 elements, there are 6 possible relationships of each pair. Since there are 21 pairs for the 7 elements, there are totally 126 possible relationships. Using the two thresholds set above, there are only 12 relationships satisfying the conditions, which are exactly the true relationships (1). There are many relationships among the 126 relationships satisfying only one condition, but not both. For example, the relationships 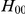 in 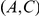 and 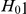 in 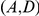 satisfy the counting number condition, but not the p-value condition; the relationships 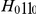 and 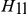 in 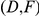 satisfy the p-value condition, but not the counting number condition. It shows that any one of the two steps is an important condition for identifying the true relationships. In this case, we can recover all true relationships using the proposed method.

Next, we implement the algorithm of Li and Lu [29] in the simulated data. Since this algorithm does not provide a specific threshold selection method, we adopt different thresholds and find that the best situation is to recover 11 relationships . In this case, one relationship 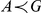 is misjudged to be 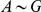.

In order to compare the counting method with Sahoo *et al.* [27], we implement their method in this simulated data. There are only two relationships 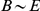 and 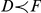 recovered from their method with “
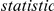
” values 4.05 and 3.18, respectively. The “
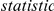
” values for relationships of other pair elements are smaller than 3, resulting in inaccuracy of identifying the other true relationships. It shows that the method of [27] is less efficient and accurate in recovering the true relationships than the counting method from the simulation study for the case with measurement error.

Beside the example with 7 elements, a more comprehensive example with a larger network (Figure S1) that shows the superiority of the proposed method is given in the supplementary material.

### Yeast expression data

We revisit the MAPK pathway example from the Introduction. The datasets used in analyzing the MAPK pathway include 81 experimental data excluding two data with missing values, 57 from Spellman *et al.* [31] and 26 from Zhu *et al.* [32] . The datasets from Spellman *et al.* [31] include 18 data from the alpha factor experiments, 14 data set from the Elutrtation experiments and 24 data sets from cdc15 experiments. The datasets from Zhu *et al.* [32] include 25 data from Forkhead experiments. The raw data can be download from the Stanford Microarray Database [33]. We adopt values corresponding to the Log(base2) column in the raw dataset to reconstruct the MAPK pathway, which are log ratio values of red to green signal.

A gene state is regarded as on state or off state when the log ratio value of red to green signal is greater than or less than 0, respectively. The gene expression data for the 81 experimental data (Table S1) are given in the supplementary material.

In this study, we apply the two-step approach to explore the expression profiles, and show exploratory results on the pathway. The results are also compared with the Li and Lu's method [29] and Shaoo *et al.* method [27].

We implement the proposed method to the yeast cell cycle data [31], [32]. In our analysis, we assume that the level 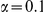. According to the threshold selection formulas (2), the thresholds for the similar and prerequisite relationships are 61 and 69, respectively. And the threshold for the asymptotical p-value we selected is 1.

According to the network structure reconstructed using our proposed approach, we can see that Wsc2p and Mid2p activate Rho1p, Pkc1p and Bck1p which results in activation of the downstream of MAPK cascade, Mkk1p and Mlp1p. Activated Wsc2p also interacts with Mid2p. The functions of genes Swi4p, Swi6p and Rlm1p in the downstream of the network are not significant in our approach.

**Figure 3:**
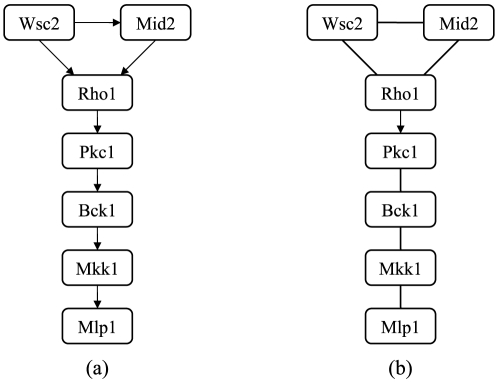
Some pairwise relationships identified by the two-steps counting approach (a), and the Li and Lu method (b) using the expression data of yeast Saccharomyces cerevisiae.

The reconstruction results of the DAB network using the two-step approach and the method of Li and Lu [29] are illustrated in Figure 3(a) and Figure 3(b), respectively. In addition, we also implemented the method of Shaoo *et al.* [27] in this real yeast data. The results show that there are no pair relationships detected by the method of Shaoo *et al.* [27], because all “
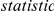
” values are smaller than 3 for any two elements. Therefore, compared with the methods in Li and Lu [29] and Shaoo *et al.* [27], our proposed method is more useful for finding the cascade relationship.

## Discussion

For the implementation of the network reconstruction algorithm, the greatest complexity lies in the computation of p-value for every two elements. The number of all pair is 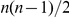 where 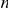 is the number of elements. Therefore, the time complexity for the proposed approach is 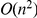 showing that the proposed method is capable of handling thousands of genes simultaneously.

This study mainly focuses on reconstructing pathway by gene expression. Although pathway reconstruction methods based on gene expression have been widely discussed in the literature, there is a limitation on the gene expression methods. A biological pathway comprises more than genetic interactions alone. Long chains of vents may happen on the protein level (e.g. (de)activation by phosphorylation) which does not necessarily have to be regulated via gene expression. Therefore, these gene expression methods can be expected to reconstruct pathways that are regulated via gene expression, but not other biological interactions.

In summary, we propose a two-step approach to test the biological pathways from noisy array data. This new method has the advantages of easy calculation for the counting numbers and simple closed forms of the p-value. From the simulation results, we can see that this method can precisely estimate the true relationships for most of the situations. Compared with the other existing methods, it can provide a more accurate and efficient alternative approach for reconstructing the biological network.

## Materials and Methods

### Appendix A: Threshold Selection

(i) Suppose the misclassification probability is 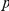. For a similar relationship such as the case 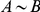, in this case, we have
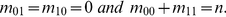
(3)


With misclassification error, the counting number corresponding to the relationship is
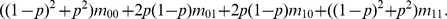



By (3), the last equation is equal to 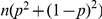, which is the mean of the counting number if this similar relationship holds. Since we cannot expect that the counting number is always equal to the mean, we look for a lower bound of the counting number as a threshold. From the viewpoint of constructing confidence interval, if 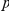 is unknown, a 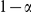 upper bound of 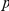 is 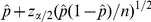, where 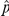 is an estimator of 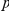 and 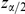 is the upper 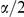 quantile of the standard normal distribution. The bound 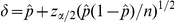 is an upper bound of 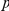. Then 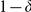 is a lower bound of 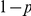. Here we replace 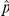 by 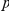 in the upper bound and suggest 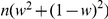, where 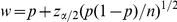 as a threshold. We expect that the counting number is greater than the threshold if the similar relationship holds. Beside using the conventional confidence interval, we can also consider some improved intervals discussed in literature [34]–[37].

(ii) Assume for two elements 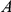 and 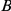, a prerequisite relationship holds. In this case, we have
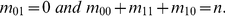
(4)


With misclassification errors, the counting number corresponding to the relationship is
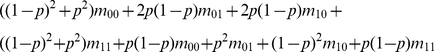
(5)


By (4), (5) is equal to
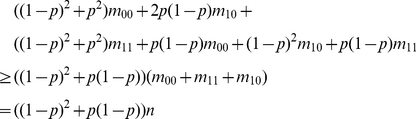
(6)


By a similar argument as in (i), we suggest 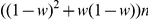 as a threshold for the prerequisite relationship.

### Appendix B: Computational details

The methods for testing the 6 hypotheses in Table 3 are listed as following.

(i) For deriving the p-value of the test:

(ii)
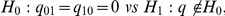
we can consider the following two different situations. Note that the condition 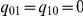 in hypothesis 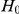 is equivalent to 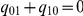 because 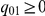 and 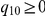.

(I) The misclassification probability 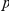 is zero.

The statistics
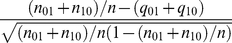
(7)has an asymptotic standard normal distribution under the null hypothesis 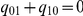.

(II) The misclassification probability 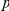 is greater than zero.

In this case, the mean and the variance of the random variable are 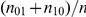 is
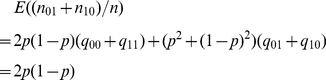
(8)and
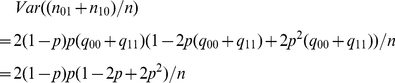
(9)under the null hypothesis.

Consequently, the asymptotic p-value is
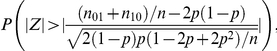
which can be rewritten as (3).

(iii) For deriving the p-value of the test:

(iv)
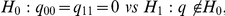
by an argument similar to (i), for 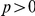, the asymptotic p-value is
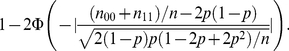
(10)


(iii) For deriving the p-value of the test:
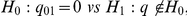
consider the case of 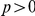.

Under the null hypoyhesis, the mean and variance of the statistics 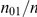 are
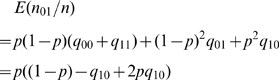
(11)and
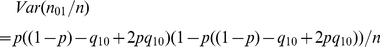
(12)under the null hypothesis.

The asymptotic p-value is
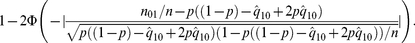
(13)


(iv) For deriving the p-value of the test:
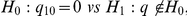
by an argument similar to (iii), the asymptotic p-value is
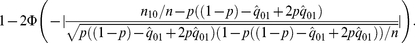
(14)


(v) For deriving the p-value of the test:
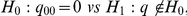
by an argument similar to (iii), the asymptotic p-value is
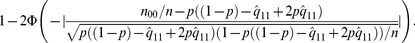
(15)


(vi) For deriving the p-value of the test:
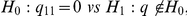
by an argument similar to (iii), the asymptotic p-value is
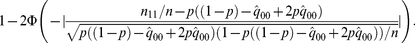
(16)


The estimators 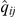 of 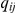 in the above formulas of asymptotic p-values are given in Appendix C.

### Appendix C: Frequency estimation

If the misclassification probability 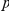 is known, the methods for estimating the probability 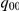, 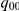, 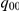 and 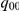 are listed as follows.

According to Table 5, we have
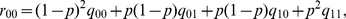


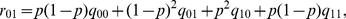


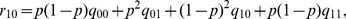


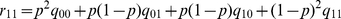



Note that 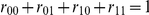 and 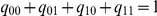. By solving the above equations, we have
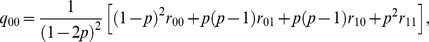


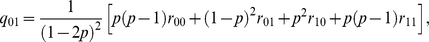


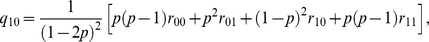


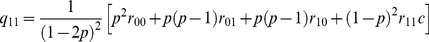



The derived values are used as estimators for 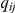.

### Appendix D: Misclassification probability estimation

If 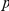 is unknown, we can apply the maximum likelihood approach to estimate 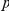. By Table 5, we can rewrite the multinomial model for the observations 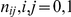 in terms of 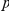 and other parameters. The maximum likelihood approach for deriving the maximum likelihood estimator of 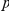 is based on the likelihood function
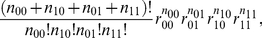
(17)where 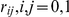. This involve 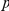 and other parameters, 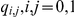, given in Appendix C. The maximum likelihood approach is to find the maximum likelihood estimators of 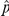 and 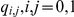 such that the estimators can maximize the likelihood function (17) [29].

## Supporting Information

Figure S1 An example of Boolean network with 10 elements.
(DOCX)

Table S1 The 81 experimental yeast expression data.
(DOCX)
