# Supplementary material for: Constructing Biological Pathways by a Two-Step Counting Approach
Source: PLoS One. 2011 Jun 1;6(6):e20074. doi: 10.1371/journal.pone.0020074 (PMC3105984; doi:10.1371/journal.pone.0020074)
Supplement: Figure S1 — An example of Boolean network with 10 elements. (DOCX) [file pone.0020074.s001.docx]

**Supporting Information**

A biological pathway with 10 elements

An example with 10 elements (Figure S1) is provided in order to show that the proposed method can be applied to a larger network.

Figure S1. An example of Boolean network with 10 elements.

Among the 1024 possible states of the 10 elements, there are only 69 compatible states satisfying Figure S1. To illustrate the concept, we list several compatible states here, which are (A, B, C, D, E, F, G, H, I, J)=(0,0,0,1,0,0,0,0,0,0),( 1,0,0,1,0,0,0,0,0,0),… Then, we generate 300 states from these 69 compatible states with a misclassification probability of 0.05. In this case, the maximum value for the counting number is 300 because the sample size is 300. We set α=0.1. According to the threshold formulas (2), the thresholds for the similar and prerequisite relationships are 262 and 280, respectively.

We use these thresholds to find and determine the relationships. By repeating the process of generating 300 states 100 times, we found that the false discovery rate of the proposed method is 0.02. We also apply Li and Lu’s method and Shaoo’s method in this example. It’s hard to use Shaoo’s method to reconstruct this pathway because it is too conservative. In addition, Li and Lu’s method does not provide a general threshold selection method and is time-consumption. It also has a higher false discovery rate than the proposed method.
