# Supplementary material for: Constructing Biological Pathways by a Two-Step Counting Approach
Source: PLoS One. 2011 Jun 1;6(6):e20074. doi: 10.1371/journal.pone.0020074 (PMC3105984; doi:10.1371/journal.pone.0020074)
Supplement: Table S1 — The 81 experimental yeast expression data. (DOCX) [file pone.0020074.s002.docx]

**Supporting Information**

**Table S1.** **The 81 experimental yeast expression data**

| *Experiment* | *WSC2* | *MID2* | *RHO1* | *PKC1* | *BCK1* | *MKK1* | *MLP1* |
| --- | --- | --- | --- | --- | --- | --- | --- |
| *Alpha factor block-release* | *0.146* | *0.82* | *0.456* | *-0.284* | *0.388* | *0.191* | *1.818* |
|  | *-0.122* | *-0.468* | *0.276* | *-0.065* | *-0.801* | *0.29* | *-1.106* |
|  | *0.602* | *0.465* | *0.202* | *-0.308* | *-0.2* | *0.584* | *-0.837* |
|  | *0.558* | *0.096* | *0.25* | *-0.139* | *0.092* | *0.251* | *-0.598* |
|  | *0.192* | *-0.113* | *0.519* | *0.139* | *-0.831* | *0.484* | *-0.378* |
|  | *-0.684* | *-0.525* | *0.112* | *0.182* | *-0.049* | *-0.16* | *-0.363* |
|  | *0.5* | *1.61* | *0.528* | *0.145* | *-0.526* | *0.223* | *-0.118* |
|  | *0.508* | *0.114* | *-0.021* | *0.166* | *-0.436* | *-0.339* | *-0.427* |
|  | *0.063* | *0.114* | *-0.196* | *0.189* | *0.064* | *-0.294* | *-0.658* |
|  | *0.776* | *0.183* |  | *-0.189* | *0.32* | *0.331* | *-0.112* |
|  | *0.757* | *0.317* | *0.351* | *-0.047* | *-0.313* | *0.246* | *-0.326* |
|  | *0.052* | *0.011* | *0.337* | *0* | *-0.354* | *0.078* | *-0.541* |
|  | *0.157* | *0.029* | *0.129* | *0.192* | *-0.108* | *-0.02* | *-0.256* |
|  | *0.046* | *0.176* | *0.186* | *0.297* | *0.184* | *-0.15* | *-0.314* |
|  | *-0.544* | *-0.391* | *0.035* | *-0.053* | *0.409* | *-0.143* | *-0.343* |
|  | *0.603* | *0.338* | *-0.188* | *0.025* | *0.08* | *-0.135* | *-0.325* |
|  | *-0.21* | *-0.095* | *0.711* | *-0.08* | *0.51* | *-0.06* | *0.7* |
|  | *-0.455* | *-0.1* | *0.244* | *0.098* | *0.022* | *0.004* | *-0.02* |
| *Cdc15* | *-0.963* | *0.073* | *0.214* | *-0.188* | *-0.823* | *0.19* | *-0.202* |
|  | *0.806* | *0.375* | *0.404* | *-0.223* | *-1.874* | *0.065* | *-0.282* |
|  | *0.985* | *0.437* | *0.672* | *-0.467* | *-0.478* | *0.201* | *-0.387* |
|  | *0.602* | *0.358* | *0.734* | *-0.357* | *-0.8* | *0.194* | *-0.501* |
|  | *0.549* | *-0.191* | *0.703* | *-0.171* | *-0.291* | *-0.045* | *-0.033* |
|  | *-0.605* | *-0.344* | *0.162* | *-0.438* | *-1.182* | *-0.215* | *-1.458* |
|  | *-0.35* | *-0.293* | *0.357* | *-0.403* | *-1* | *-0.163* | *0.143* |
|  | *0.208* | *1.147* | *0.627* | *-1.002* | *0.174* | *-0.313* | *-0.307* |
|  | *-0.806* | *0.968* | *0.314* | *-0.479* | *-0.171* | *-0.166* | *-0.568* |
|  | *0.015* | *0.692* | *0.085* | *-0.266* | *0.162* | *-0.43* | *0.037* |
|  | *0.913* | *0.971* | *0.489* | *-0.538* | *0.476* | *-0.34* | *-0.178* |
|  | *0.818* | *0.41* | *0.504* | *-1.115* | *-0.807* | *-0.752* | *-1.438* |
|  | *0.054* | *0.62* | *0.545* | *-0.595* | *1.692* | *-0.215* | *-0.852* |
|  | *-1.862* | *-0.417* | *0.063* | *-0.341* | *0.074* | *-0.052* | *-0.624* |
|  | *-1.187* | *0.305* | *0.079* | *-1.121* |  | *-0.739* | *-1.821* |
|  | *-0.544* | *-0.006* | *-0.11* | *0.011* | *-0.558* | *-0.221* | *-0.215* |
|  | *0.806* | *0.025* | *0.519* | *-0.588* | *0.101* | *-0.229* | *-2.204* |
|  | *0.5* | *0.071* | *0.135* | *-0.821* | *-5.833* | *-0.298* | *-4.838* |
|  | *-0.617* | *-0.261* | *-0.01* | *-0.391* | *-1.521* | *-0.675* | *-0.339* |
|  | *-0.204* | *-0.118* | *0.615* | *-0.69* | *-0.084* | *-0.14* | *-0.279* |
|  | *-0.177* | *-0.033* | *0.664* | *-0.565* | *0.734* | *-0.06* | *-0.594* |
|  | *-0.684* | *0.131* | *0.386* | *-0.067* | *-0.351* | *0.142* | *-0.804* |
|  | *0.931* | *-0.208* | *0.798* | *-0.579* | *0.067* | *0.097* | *0.742* |
|  | *-0.883* | *0.875* | *0.308* | *-0.408* | *0.816* | *-0.374* | *0.243* |
| *Elutration* | *-1.577* | *0.333* | *0.232* | *0.511* | *-0.643* | *-0.261* | *0.049* |
|  | *-1.452* | *-0.684* | *-0.027* | *-0.174* | *0.003* | *-0.026* | *-0.3* |
|  | *-0.881* | *-0.403* | *0.012* | *0.141* | *0.028* | *-0.129* | *-0.077* |
|  | *0.25* | *0.063* | *0.004* | *-0.085* | *-0.173* | *0.251* | *-0.303* |
|  | *0.52* | *0.553* | *0.303* | *-0.159* | *0.202* | *-0.003* | *-0.468* |
|  | *0.711* | *0.75* | *0.198* | *-0.197* | *0.358* | *0.208* | *0.308* |
|  | *1.005* | *0.048* | *0.708* | *0.026* | *0.017* | *0.152* | *0.025* |
|  | *0.64* | *0.046* | *0.363* | *0.185* | *0.302* | *0.043* | *-0.012* |
|  | *0.479* | *0.223* | *0.167* | *0.043* | *0.089* | *0.143* | *-0.596* |
|  | *0.744* | *0.087* | *0.471* | *0.419* | *0.563* | *0.082* | *0.284* |
|  | *0.246* | *-0.14* | *0.465* | *0.299* | *0.161* | *0.004* | *0.477* |
|  | *0.371* | *-0.118* | *0.449* | *0.547* | *0.511* | *0.155* | *-0.562* |
|  | *-0.163* | *-0.624* | *0.436* | *0.135* | *0.242* | *-0.367* | *-0.415* |
|  | *-0.975* | *-0.516* | *0.02* | *0.062* | *-0.13* | *-0.029* | *-0.122* |
| *Forkhead* | *0.351* | *0.355* | *0.112* | *-0.171* | *-0.307* | *0.363* | *-0.122* |
|  | *0.078* | *0.257* | *-0.141* | *0.096* | *0.019* | *-0.371* | *-0.479* |
|  | *-0.093* | *0.203* | *-0.309* | *0.267* | *-0.021* | *-0.02* | *-0.373* |
|  | *0.118* | *0.065* | *0.037* | *-0.224* | *0* | *-0.32* | *0.197* |
|  | *0.755* | *-0.117* | *0.222* | *0.168* | *0* | *-0.246* | *-0.155* |
|  | *0.49* | *-0.266* | *-0.137* | *0.036* | *-0.02* | *-0.156* | *0.152* |
|  | *0.197* | *-0.024* | *-0.235* | *0.433* | *0.377* | *-0.193* | *-0.302* |
|  | *0.363* | *0.168* | *-0.484* | *0.534* | *0.281* | *0.047* | *-0.592* |
|  | *-0.158* | *-0.165* | *-0.221* | *0.51* | *0.456* | *0.062* | *-0.165* |
|  | *-0.156* | *-0.085* | *-0.41* | *0.318* | *0.309* | *-0.171* | *-0.183* |
|  | *-0.116* | *0.504* | *-0.24* | *0.399* | *0.413* | *-0.053* | *-0.259* |
|  | *-0.262* | *0.031* | *-0.436* | *0* | *0.096* | *-0.367* | *-0.885* |
|  | *0.473* | *-0.228* | *0.625* | *-0.289* | *-0.124* | *0.185* | *2.111* |
|  | *0.48* | *-0.146* | *-0.046* | *-0.073* | *-0.032* | *-0.132* | *1.65* |
|  | *0.931* | *0.387* | *0.402* | *-0.242* | *0.065* | *0.291* | *0.493* |
|  | *0.38* | *0.305* | *-0.084* | *0.321* | *0.087* | *-0.123* | *0.75* |
|  | *0.061* | *0.141* | *-0.175* | *-0.065* | *-0.031* | *-0.008* | *1.893* |
|  | *0.058* | *-0.095* | *-0.261* | *-0.339* | *-0.079* | *-0.185* | *0.843* |
|  | *-0.385* | *0.124* | *-0.343* | *-0.155* | *0.037* | *-0.095* | *1.715* |
|  | *0.149* | *0.654* | *-0.353* | *-0.097* | *0.035* | *0.221* | *1.551* |
|  | *0.39* | *0.472* | *-0.306* | *-0.066* | *0.027* | *-0.019* | *1.441* |
|  | *0.541* | *0.599* | *0.051* | *0.143* | *-0.168* | *-0.099* | *1.796* |
|  | *0.645* | *0.25* | *0.179* | *-0.045* | *0.038* | *-0.098* | *0.945* |
|  | *0.772* | *0.315* | *0.064* | *-0.028* | *-0.024* | *-0.153* | *0.367* |
|  | *0.164* | *0.111* | *-0.385* | *0.075* | *0.052* | *-0.137* | *-0.817* |
